# Characterizing *Ancylostoma caninum *transcriptome and exploring nematode parasitic adaptation

**DOI:** 10.1186/1471-2164-11-307

**Published:** 2010-05-14

**Authors:** Zhengyuan Wang, Sahar Abubucker, John Martin, Richard K Wilson, John Hawdon, Makedonka Mitreva

**Affiliations:** 1 The Genome Center, Department of Genetics, Washington University School of Medicine, St. Louis, MO 63110, USA; 2 Department of Microbiology and Tropical Medicine, George Washington University Medical Center, Washington, DC 20037, USA

## Abstract

**Background:**

Hookworm infection is one of the most important neglected diseases in developing countries, with approximately 1 billion people infected worldwide. To better understand hookworm biology and nematode parasitism, the present study generated a near complete transcriptome of the canine hookworm *Ancylostoma caninum *to a very high coverage using high throughput technology, and compared it to those of the free-living nematode *Caenorhabditis elegans *and the parasite *Brugia malayi*.

**Results:**

The generated transcripts from four developmental stages, infective L3, serum stimulated L3, adult male and adult female, covered 93% of the *A. caninum *transcriptome. The broad diversity among nematode transcriptomes was confirmed, and an impact of parasitic adaptation on transcriptome diversity was inferred. Intra-population analysis showed that *A. caninum *has higher coding sequence diversity than humans. Examining the developmental expression profiles of *A. caninum *revealed major transitions in gene expression from larval stages to adult. Adult males expressed the highest number of selectively expressed genes, but adult female expressed the highest number of selective parasitism-related genes. Genes related to parasitism adaptation and *A. caninum *specific genes exhibited more expression selectivity while those conserved in nematodes tend to be consistently expressed. Parasitism related genes were expressed more selectively in adult male and female worms. The comprehensive analysis of digital expression profiles along with transcriptome comparisons enabled identification of a set of parasitism genes encoding secretory proteins in animal parasitic nematode.

**Conclusions:**

This study validated the usage of deep sequencing for gene expression profiling. Parasitic adaptation of the canine hookworm is related to its diversity and developmental dynamics. This comprehensive comparative genomic and expression study substantially improves our understanding of the basic biology and parasitism of hookworms and, is expected, in the long run, to accelerate research toward development of vaccines and novel anthelmintics.

## Background

Genomic data is revolutionizing molecular parasitology and has been used to prioritize drug targets in parasites at a genomic level [1]. Similarly, pan-phylum genomic studies in parasitic nematodes have identified both highly conserved nematode-specific proteins [2], which are attractive as drug candidates as their targeting will not affect the host, and nematode-specific indels in essential proteins [3], which could also be good candidates for "indel-based" drug design in nematodes. Complementing the genomic data, expression data reflects the dynamics of genetic information. Analysis of digital expression data, obtained by sequencing cDNAs, is crucial for studying and understanding organism's development, physiology, and environmental adaptation. Knowledge of these mechanisms in parasites is essential to substantially accelerate research toward the development of both new therapies to prevent parasite infections and vaccines (or novel anthelmintics) needed to control them.

Infection of humans by parasitic nematodes results in substantial human mortality and morbidity, especially in tropical regions of Africa, Asia, and the Americas. Hookworms, probably the most significant public health threat of these nematodes, are the second largest contributor to the 26.7 million annual DALYs (Disability Adjusted Life Years) from iron-deficiency anemia due to blood feeding by adult worms. Chronic anemia from hookworm infection is particularly devastating to children, who suffer from stunted growth and impaired intellectual development, to mothers who are at increased risk for anemia during pregnancy and childbirth, and to the elderly [4-6]. Current hookworm control strategies are limited to deworming of infected people using anthelmintic drugs. However, rapid re-infection in endemic areas and the lack of sterile immunity necessitates repeated treatments, which will in turn result in resistance. The high rates of re-infection after drug therapy mean that vaccines remain the best hope for worm control in humans in the future. No vaccine is yet available, despite substantial support from The Bill and Melinda Gates Foundation specifically for the development of a hookworm vaccine [7]. Until safe and effective vaccines are developed, anthelmintics will continue to be used for treatment and control of nematode infections in humans. Thus, there is a critical need for further research to identify new vaccine and drug targets which requires better understanding of the biology and parasitism of these devastating parasites.

*Ancylostoma caninum*, a canine hookworm closely related to the human parasites *Ancylostoma duodenale *and *Necator americanus *[8], is the most widely used model for human hookworm infections [9]. Similar to other hookworms, adult *A. caninum *inhabit the small intestine and produce eggs that pass in the feces and hatch in the soil. The first stage larva feeds on bacteria and molts twice to form the non-feeding, infective third stage (iL3). iL3 enters the host by penetrating the skin, molts twice, and matures in to the adult (Ad) stage in the small intestine. *A. caninum *iL3 can also infect a host, temporarily abort maturation and enter an arrested state (hypobiosis) within the host's somatic tissues [10], reactivating in response to host physiological changes such as pregnancy [11].

*A. caninum *is a Clade V nematode [12] that also includes the well-studied free-living model nematode *Caenorhabditis elegans. C. elegans *was the first multicellular genome to be sequenced [13] and it remains the only metazoan for which the sequence of every nucleotide is known to high confidence. Recently, the genome of the human parasite *Brugia malayi*, has been sequenced and analyzed [14]. *Brugia malayi *is phylogenetically classified in Clade III [15]. The distant phylogenetic relationship between *A. caninum *and *B. malayi *(compared to *A. caninum *and *C. elegans*) makes investigation of nematode adaptation to parasitism easier, as similarities shared by *A. caninum *and *B. malayi *(but not by *C. elegans*) are likely to be associated with adaptation to parasitism. Our previous studies based on limited coverage of *A. caninum *revealed the existence of genes unique to hookworm and the different selective pressures on these genes [16,17]. Another study using microarray technology [18] found several hundred genes in *A. caninum *changed their expression during the worm's transition from a free living to a parasitic larva. However, because of the limitation of the data and/or methods of these studies, many questions, especially those related to parasitism, remain to be fully explored.

To better understand the biology of parasitism and facilitate prioritization of potential vaccine and drug targets, the present study deeply sequenced the *A. caninum *transcriptome with a combination of two distinct sequencing technologies, ABI Sanger capillary and 454/Roche massively parallel sequencing platforms. Over 1.5 million cDNAs were generated from different cDNA libraries constructed from pre-parasitic, parasitic larval and adult stages. These reads covered over 90% of the *A. caninum *transcriptome with an average depth of 10×. This dataset was also used to perform comprehensive comparative analysis among *A. caninum, B.malayi *and * C. elegans*, and the unprecedented depth of coverage enabled comparison of digital expression profiles leading to reliable identification of differentially expressed genes during development. This study provides the first nearly complete transcriptome from a parasitic nematode and provides valuable information about nematode adaptations to parasitism, in addition to revealing several candidates for further study as drug target or vaccine components.

## Results

### Sequence acquisition, organization and transcriptome coverage

Over 1.5 million ESTs were generated from 4 stages, infective L3 larva (iL3), activated L3 larva (ssL3), adult male (M), and female (F), of *A. caninum *(Table 1). These 1,567,105 reads include 1,483,002 pyrosequencing reads (Roche/454 reads, average length 232 bases) and 84,103 Sanger reads (average length 748 bases). The larval stages were represented by nearly half a million reads, and the adult stages with nearly 300,000 reads (Table 1).

**Table 1:** Sequence characteristics

	Reads (#)					
			
	infective L3	serum stimulated L3	Female	Male	Total reads	Mean Length (bp)
Roche/454FLX	474,766	458,249	277,319	272,668	1,483,002	232
ABI Sanger	23,429	21,722	18,960	19,992	84,103	748
						
Total	498,195	479,971	296,279	292,660	1,567,105	-

Assembly, which was performed to reduce data redundancy and improve sequence quality and length, grouped the sequences into 48,326 transcripts longer than 90 bp, for a total of 23 Mb. The transcript consensus sequences are available at http://nematode.net[19]. The average transcript length was 477 bp and average coverage was 10×. Using the core eukaryotic genes as a reference, we estimated that 93% of the *A. caninum *transcriptome is identified (*See *additional file 1), making this the first parasitic nematode with a near complete sequenced transcriptome.

### Nematode transcriptome diversity and parasitism related genes

**Figure 1:**
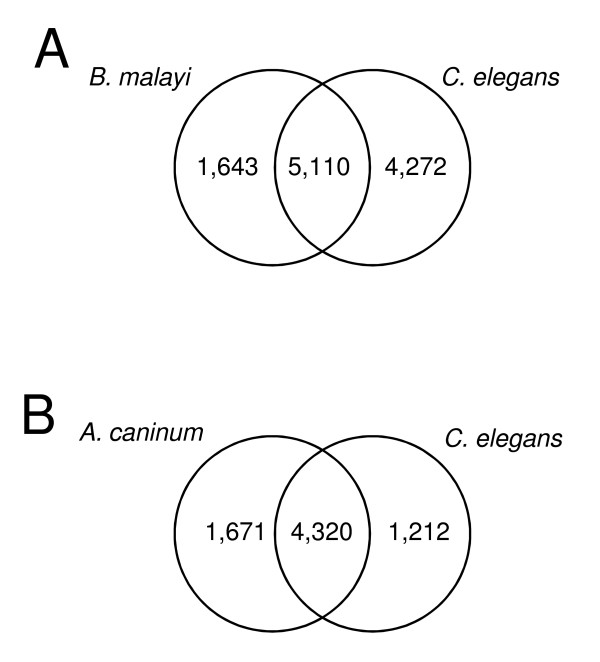
**Venn diagram showing distribution of BLAST matches**. Amino acid level homologies with bitscore of 50 or better were considered. (**A**) *A. caninum *transcripts homologous to *B. malayi *and *C. elegans*. Only 23% of the transcripts (11,025/48,326) shared homology, leaving 37,301 transcripts to be specific to *A. caninum*. (**B) ***B. malayi *genes homologous to *A. caninum *and *C. elegans*. About 62% of the 11,609 *B. malayi *genes shared homology. Higher number of *B. malayi *genes had homologs to the parasitic *A. caninum *compared to the free-living *C. elegans*, and 4,406 *B. malayi *genes did not share homology.

Even though *A. caninum *and *C. elegans *fall in the same phylogenetic Clade (Clade V)[12], only about 20% of *A. caninum *transcripts are homologous to *C. elegans *coding genes, and even lower number (14%) to *B. malayi *coding genes (Figure 1A). However, when only considering the highly expressed transcripts (those sequenced deeply enough to provide confident stage selectivity in this case) about 43% of *A.caninum *transcripts are homologous to *C. elegans*. When all the transcripts were considered, the vast majority (77%) of the *A. caninum *transcripts were species-specific. This indicates high transcriptome diversity among nematodes. However, this diversity did not correspond to a drastic difference on functional level. The total unique number of KOs associated to the *A. caninum *and *C. elegans genes *were very similar (Table 2), with only one (amino acid metabolism pathway; P < 0.001) out of the 33 identified pathways having a statistically significant increased number of unique KOs (359 vs. 315) in *A. caninum*.

**Table 2:** KEGG pathway mappings for *A. caninum *and *C. elegans *orthologs

Major Category	Pathway	A.caninum KOs	C.elegans KOs	Total KO in the pathway
1. Metabolism		1467	1386	2230
	1.1 Carbohydrate Metabolism	357	327	547
	1.2 Energy Metabolism	255	247	406
	1.3 Lipid Metabolism	234	237	332
	1.4 Nucleotide Metabolism	118	122	165
	1.5 Amino Acid Metabolism	359	315	474
	1.6 Metabolism of Other Amino Acids	86	85	125
	1.7 Glycan Biosynthesis and Metabolism	113	119	158
	1.8 Biosynthesis of Polyketides and Nonribosomal Peptides	4	4	4
	1.9 Metabolism of Cofactors and Vitamins	180	173	297
	1.10 Biosynthesis of Secondary Metabolites	47	46	57
	1.11 Xenobiotics Biodegradation and Metabolism	122	115	180
2. Genetic Information Processing		489	513	793
	2.1 Transcription	70	77	85
	2.2 Translation	149	145	175
	2.3 Folding, Sorting and Degradation	191	213	368
	2.4 Replication and Repair	84	83	174
3. Environmental Information Processing		1049	1106	1846
	3.1 Membrane Transport	120	140	391
	3.2 Signal Transduction	555	571	919
	3.3 Signaling Molecules and Interaction	462	483	713
4. Cellular Processes		886	912	1176
	4.1 Cell Motility	162	165	203
	4.2 Cell Growth and Death	172	181	246
	4.3 Cell Communication	231	234	250
	4.4 Endocrine System	239	247	281
	4.5 Immune System	260	267	393
	4.6 Nervous System	62	62	66
	4.7 Sensory System	31	34	38
	4.8 Development	99	99	102
	4.9 Behavior	7	8	10

There were 1,643 transcripts with *B. malayi *homologs (1,365 genes) but no *C. elegans *homologs (Figure 1A) despite *A. caninum *being more closely related phylogenetically to *C. elegans*. The majority of these transcripts (1,093 out of 1,643) failed to find any GO annotations. Nevertheless, functions of the 550 transcripts having GO annotation are enriched in 3 GO terms, prolyl oligopeptidase activity (GO:0004287, P = 3.5e-6), nucleic acid binding (GO: 0003676, P = 5.1e-5), and DNA binding (GO: 0003677, P = 1.7e-3), with the most enriched category being prolyl oligopeptidase activity. In addition, malic enzyme activity was enriched (P = 5.2e-3) though it failed our FDR cutoff because of the small number of entries in this activity. As a comparison, no GO term enrichment was detected when considering the *B. malayi *genes with homology to *C. elegans *but not *A. caninum*. Meanwhile, homology comparison among the free-living *C. elegans *and the parasites *A. caninum *and *B. malayi *found that more *B. malayi *genes share homology with *A. caninum *(5,991) than with *C. elegans *(5,532) (Figure 1B). The higher number of homologous genes among parasites was statistically significant (P < 1.0e-4, Chi-square test). Since *B. malayi *(Clade III) is phylogentically more distant from *A. caninum *than *C. elegans *(both are in Clade V) [12], *B. malayi *would share a similar level of homology with both *C. elegans *and *A. caninum *if parasitism had no effect on gene evolution. Therefore, we hypothesize that the 1,643 transcripts represent putative parasitism related genes.

### Comparative genomics of gene expression during development of *A. caninum*

**Figure 2:**
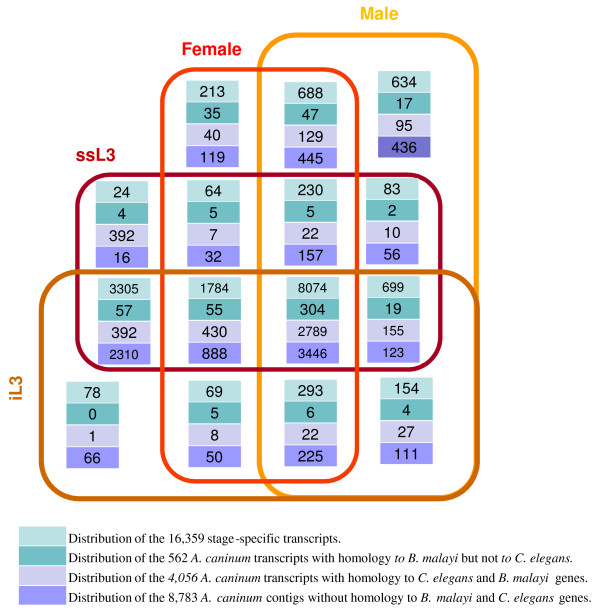
**Distribution of *A. caninum *transcripts based on stage or origin of each read**. iL3: infective L3; ssL3: serum stimulated L3.

The deep sequencing of this study allowed us to examine differential expression of inferred transcripts and shed light on their functions due to the association of the gene expression with molecular function. Figure 2 summarizes the expression selectivity of the 16,359 transcripts that have sequencing depth for confident estimation of expression selectivity, and Table 3 contains the ten most abundant transcripts selectively expressed in different stages (The list of the top 20 most abundant transcripts is available as additional file 2). Only half of the transcripts are expressed through all the stages. The most dramatic change observed between the developmental stages studied was the transition from larvae to adult: more than 3,000 transcripts were turned off and nearly 1,500 turned on. This is not unexpected, especially since the developing L4 stage was not examined. The serum stimulation turned off 78 transcripts, and turned on 401 transcripts. More than one third of the turned-on genes were turned off again in M, F, or M and F. Consistent with these expression changes, comparison of the preparasitic to parasitic (iL3 vs. ssL3) and parasitic larval to adult stages (ssL3 vs. M + F) revealed that nearly twice the number of transcripts have significant expression changes in the latter transition than the former (*See *additional file 3).

**Table 3:** The most abundantly represented transcripts in the *A. caninum *cDNA library expressed genes

Stage	Contig id	Female	Male	iL3	ssL3	Total	GO id	Go descriptor
Female specific								
	contig00905	168	0	0	0	168		-
	contig03165	118	0	0	0	118	GO:0004252	^a^serine-type endopeptidase activity
	contig05541	103	0	0	0	103	GO:0005529	^a^sugar binding
	contig02012	94	0	0	0	94	GO:0005529	^a^sugar binding
	contig05749	93	0	0	0	93		-
	contig05507	87	0	0	0	87		-
	contig05999	86	0	0	0	86		-
	contig06009	84	0	0	0	84		-
	contig02190	84	0	0	0	84		-
	contig06122	84	0	0	0	84		-
Male specific								
	contig43465	0	408	0	0	408	GO:0003676	^a^nucleic acid binding
	contig01761	0	301	0	0	301		-
	contig53299	0	294	0	0	294		-
	contig39375	0	247	0	0	247	GO:0003723	^a^RNA binding
	contig50336	0	227	0	0	227		-
	contig54027	0	213	0	0	213	GO:0006879	^b^cellular iron ion homeostasis
	contig53611	0	191	0	0	191		-
	contig40384	0	190	0	0	190		-
	contig45190	0	190	0	0	190		-
	contig51482	0	182	0	0	182		-
Adult specific (M and F)								
	contig49608	71	189	0	0	260	GO:0004531	^a^deoxyribonuclease II activity
	contig47068	101	144	0	0	245	GO:0004531	^a^deoxyribonuclease II activity
	contig51608	96	133	0	0	229	GO:0006508	^b^proteolysis
	contig49469	76	141	0	0	217	GO:0006508	^b^proteolysis
	contig41687	76	124	0	0	200	GO:0006508	^b^proteolysis
	contig52673	129	69	0	0	198		-
	contig45734	176	22	0	0	198	GO:0008289	^a^lipid binding
	contig41787	105	92	0	0	197	GO:0005576	^c^extracellular region
	contig45747	39	157	0	0	196	GO:0005576	^c^extracellular region
	contig09753	68	120	0	0	188	GO:0006508	^b^proteolysis
IL3 specific								
	contig20982	0	0	88	0	88		-
	contig53045	0	0	84	0	84	GO:0004129	^a^cytochrome-c oxidase activity
	contig46922	0	0	82	0	82		-
	contig48104	0	0	76	0	76		-
	contig54731	0	0	75	0	75	GO:0004129	^a^cytochrome-c oxidase activity
	contig20212	0	0	73	0	73		-
	contig52851	0	0	60	0	60	GO:0004129	^a^cytochrome-c oxidase activity
	contig01666	0	0	54	0	54	GO:0005506	^a^iron ion binding
	contig22515	0	0	46	0	46		-
	contig41986	0	0	43	0	43		-
ssL3 specific								
	contig43707	0	0	0	59	59		-
	contig54118	0	0	0	57	57		-
	contig21715	0	0	0	55	55		-
	contig42564	0	0	0	53	53		-
	contig47041	0	0	0	29	29	GO:0008152	^b^metabolic process
	contig29443	0	0	0	25	25	GO:0005525	^a^GTP binding
	contig33913	0	0	0	24	24	GO:0005576	^c^extracellular region
	contig43357	0	0	0	24	24	GO:0005576	^c^extracellular region
	contig43120	0	0	0	23	23	GO:0005576	^c^extracellular region
	contig17582	0	0	0	23	23		-
Larval specific (iL3 and ssL3)								
	contig03786	0	0	465	200	665		-
	contig22000	0	0	331	119	450	GO:0004289	^a^subtilase activity
	contig00904	0	0	211	237	448		-
	contig20229	0	0	207	224	431	GO:0003723	^a^RNA binding
	contig46936	0	0	232	175	407	GO:0003779	^a^actin binding
	contig43418	0	0	285	104	389		-
	contig03955	0	0	274	111	385	GO:0003735	^a^structural constituent of ribosome
	contig52848	0	0	234	148	382		-
	contig02679	0	0	138	232	370	GO:0004190	^a^aspartic-type endopeptidase activity
	contig50665	0	0	221	146	367		-
Constant expression								
	contig54178	3	1148	13	11	1175		-
	contig53913	22	837	35	66	960	GO:0008137	^a^NADH dehydrogenase (ubiquinone) activity
	contig50938	362	7	382	191	942	GO:0005215	^a^transporter activity
	contig26411	105	359	166	200	830	GO:0003824	^a^catalytic activity
	contig51127	174	385	154	94	807	GO:0005622	^c^intracellular
	contig44592	170	36	264	286	756	GO:0005515	^a^protein binding
	contig49300	138	222	241	154	755	GO:0004365	^a^glyceraldehyde-3-phosphate dehydrogenase (phosphorylating) activity
	contig43896	257	64	199	229	749	GO:0004013	^a^adenosylhomocysteinase activity
	contig40294	195	197	171	139	702	GO:0004611	^a^phosphoenolpyruvate carboxykinase activity
	contig40430	135	144	225	190	694	GO:0031419	^a^cobalamin binding

^a ^Molecular Function; ^b ^Biological Processes; ^c ^Cellular Component

Among the 401 transcripts turned on by serum stimulation, 13 of them are parasitism related (Figure 2). Given that there are a total of 562 parasitism related transcripts among all the 16,359 transcripts (whose expression selectivity could be confidently ascertained), there is no evidence to support that serum stimulation triggers an extensive expression of parasitism related genes. However, parasitism related genes are more selective expressed in adults. Nearly 18% of these genes (99 out of 562) exhibit M, F, or M and F selectivity, which is significant (P s < 1.0e-4, Chi-square test) when compared to the overall of 9% (1,505 out of 16,359; Figure 2). Interesting, compared to the small fraction of male specific transcripts related to parasitism (17/604), a large fraction of female specific transcripts (35 out of 213) are parasitism related.

The majority of nematode conserved transcripts (2,789 of 4,056) exhibited constant expression over all stages, while less than 40% (3,446 of 8,783) of the *A. caninum *specific transcripts exhibited the same expression pattern (Figure 2). This difference is highly significant statistically (P < 1.0E-10, Chi-square test). More than 80% of iL3 selective transcripts (66 out of 78) are *A. caninum *specific. The different expression pattern of conserved transcripts and *A. caninum *specific transcripts suggests caution in using cDNA data to estimate transcriptome diversity. Using limited number of cDNA reads can underestimate the diversity. For example, the homolog rate between *A. caninum *and *C. elegans *would be 54% ((8074-3446-304)/8074) if only the transcripts expressed constantly across the life cycle were considered while that is 20% when all transcripts are included.

The expression profiles defined by our sequencing were compared to the data published by Datu et al., [18]. Datu et al. studied transcriptional changes in the hookworm, *A. caninum*, during the transition from a free-living to a parasitic larva using suppression subtractive hybridization (SSH) and custom oligonucleotide microarray printed with the SSH expressed sequence tags. Comparison of the two expression profiles of the most highly up-regulated mRNAs associated with serum stimulation obtained by different orthogonal approaches confirmed consistency in expression of 9 out of the 10 up-regulated mRNA associated with serum stimulation. The only one not consistent is cDNA that has been broken into several transcripts in our assembly, therefore giving rise to this discrepancy (additional file 3).

### Functional profile of transcripts with different expression selectivity

**Figure 3:**
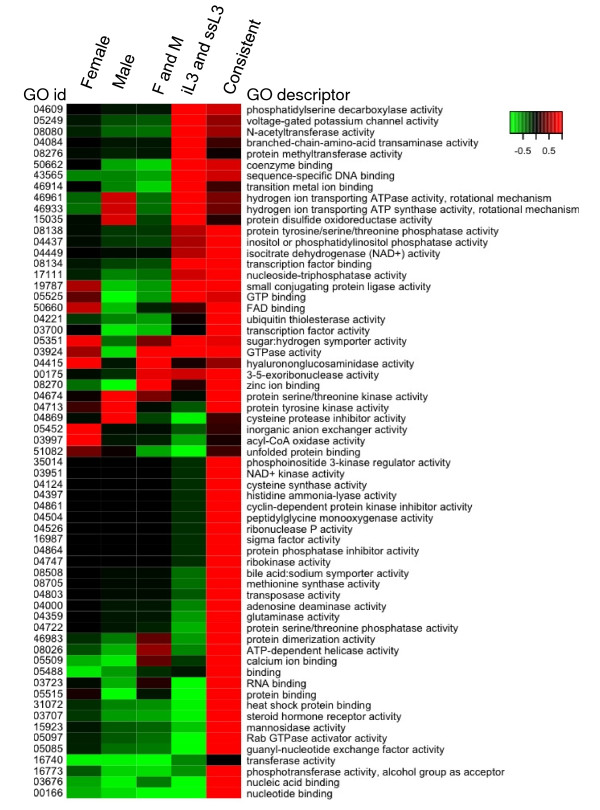
**Enriched *A. caninum *GO terms of differentially expressed transcripts**. F: female; M: male; iL3: infective L3; ssL3: serum stimulated L3.

**Figure 4:**
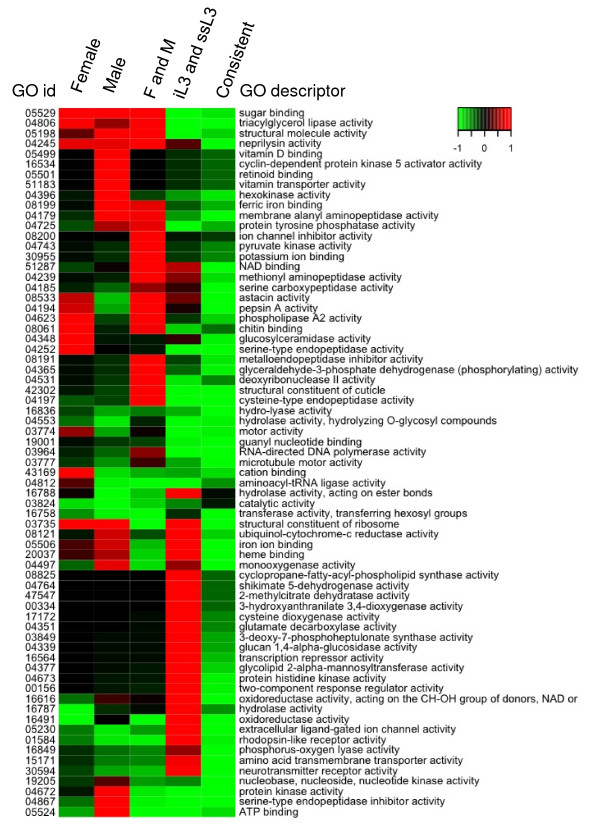
**Depleted *A. caninum *GO terms of differentially expressed transcripts**. F: female; M: male; iL3: infective L3; ssL3: serum stimulated L3.

Examining the function of transcripts with different expression patterns using GO terms revealed that enriched and depleted GO terms correlate to the biology of the corresponding stages. The heatmaps of molecular function GO terms (Figure 3 and Figure 4; See additional file 4) shows that transcripts with different expression selectivity exhibit different functional profiles, although that of the transcripts selectively expressed in both iL3 and ssL3 is closer to that of those constantly expressed, and those of the transcripts selectively expressed in F, M, and both F and M look more similar. Significantly enriched GO terms in the category of molecular functions are shown in Table 4. The top three enriched GO terms of transcripts expressed constantly over all stages are zinc ion binding (GO:0008270, P = 7.4e-12), protein binding (GO:0005515, P = 1.8e-11), and nucleic acid binding (GO:0003676, P = 9.3e-11). The top three transcripts selectively expressed in both F and M are astacin activity (GO:0008533, P = 8.8e-17), a structural constituent of cuticle (GO:0042302, P = 3.5e-8), and cysteine-type endopeptidase activity (GO:0004197, P = 4.0e-11). These terms have all been previously associated with genes involved in parasitism [20-22]. The three most significant enriched GO terms of the selectively expressed transcripts in both iL3 and ssL3 are rhodopsin-like receptor activity (GO:0001584, P = 3.1e-9), N-acetyltransferase activity (GO:0008080, P = 6.6e-6), and sugar hydrogen symporter activity (GO:0005351, P = 4.5e-5). We were only able to detect 2 significant terms from the transcripts exclusively expressed in F: serine-type endopeptidase activity (GO:0004252, P = 3.8e-4) and glucosylceramidase activity (GO:0004348, P = 6.6e-11). Interesting, the most significant enriched GO term of the transcripts exclusively expressed in M is serine-type endopeptidase inhibitor activity (GO:0004867, P = 2.6e-8).

**Table 4:** Enriched GO terms associated with transcripts with differential expression patterns

Stage	GO id*	GO descriptor	A. caninum trancripts
Female & Male			
	GO:0003674	Molecular Function	198
	GO:0004194	pepsin A activity	7
	GO:0004197	cysteine-type endopeptidase activity	16
	GO:0004245	neprilysin activity	6
	GO:0004365	glyceraldehyde-3-phosphate dehydrogenase (phosphorylating) activity	4
	GO:0004415	hyalurononglucosaminidase activity	2
	GO:0004531	deoxyribonuclease II activity	4
	GO:0004623	phospholipase A2 activity	3
	GO:0004806	triacylglycerol lipase activity	8
	GO:0005529	sugar binding	7
	GO:0008270	zinc ion binding	22
	GO:0008533	astacin activity	19
	GO:0042302	structural constituent of cuticle	7
	GO:0051287	NAD binding	6
Female			
	GO:0003674	Molecular Function	52
	GO:0004252	serine-type endopeptidase activity	4
	GO:0004348	glucosylceramidase activity	5
Male			
	GO:0003674	Molecular Function	168
	GO:0003735	structural constituent of ribosome	27
	GO:0004396	hexokinase activity	4
	GO:0004672	protein kinase activity	23
	GO:0004674	protein serine/threonine kinase activity	15
	GO:0004867	serine-type endopeptidase inhibitor activity	13
	GO:0005198	structural molecule activity	35
Infective L3 & serum stimulated L3			
	GO:0003674	Molecular Function	1388
	GO:0001584	rhodopsin-like receptor activity	43
	GO:0003735	structural constituent of ribosome	89
	GO:0003849	3-deoxy-7-phosphoheptulonate synthase activity	4
	GO:0003924	GTPase activity	23
	GO:0004377	glycolipid 2-alpha-mannosyltransferase activity	4
	GO:0004673	protein histidine kinase activity	5
	GO:0005249	voltage-gated potassium channel activity	8
	GO:0005351	sugar:hydrogen symporter activity	14
	GO:0005525	GTP binding	57
	GO:0008080	N-acetyltransferase activity	14
	GO:0015035	protein disulfide oxidoreductase activity	7
	GO:0016491	oxidoreductase activity	206
	GO:0016564	transcription repressor activity	4
	GO:0016616	oxidoreductase activity, acting on the CH-OH group of donors, NAD or NADP as acceptor	27
	GO:0016829	lyase activity	43
	GO:0020037	heme binding	35
	GO:0050662	coenzyme binding	44
Infective L3			
	GO:0003674	Molecular Function	20
	GO:0004129	cytochrome-c oxidase activity	3
	GO:0005507	copper ion binding	3
	GO:0020037	heme binding	3
Serum stimulated L3			
	GO:0003674	Molecular Function	8
	GO:0004806	triacylglycerol lipase activity	2
All stages			
	GO:0003674	Molecular Function	3052
	GO:0003676	nucleic acid binding	558
	GO:0003700	transcription factor activity	85
	GO:0003707	steroid hormone receptor activity	30
	GO:0003723	RNA binding	96
	GO:0003924	GTPase activity	44
	GO:0004000	adenosine deaminase activity	6
	GO:0004674	protein serine/threonine kinase activity	95
	GO:0004713	protein tyrosine kinase activity	77
	GO:0005515	protein binding	381
	GO:0008026	ATP-dependent helicase activity	33
	GO:0008138	protein tyrosine/serine/threonine phosphatase activity	12
	GO:0008270	zinc ion binding	258
	GO:0008508	bile acid:sodium symporter activity	4
	GO:0008705	methionine synthase activity	4
	GO:0017111	nucleoside-triphosphatase activity	188
	GO:0019787	small conjugating protein ligase activity	39
	GO:0031072	heat shock protein binding	23
	GO:0046983	protein dimerization activity	19

* Only Go terms with more than 50 hits to Mol. Function are presented.

Cut-offs: FRD < 0.1; hypogeometrics test P < 0.05.

### Secretory parasitism related genes in *A. caninum*

Next we attempted to identify transcripts encoding secreted proteins associated with parasitism. Of the 562 transcripts whose differential expression we were able to define (the top 60 differentially expressed *A. caninum *transcript with homology to *B. malayi *but not *C. elegans *are available as additional file 5), 112 were not expressed in the preparasitic iL3 stage but expressed in other stages (Figure 2), suggesting a potential role in parasitism. However, we were only able to detect a secretory signal peptide in 9 transcripts (*See *additional file 6). This low number might underestimate the true number due to the fragmented nature of our data. Another possibility is that some secretory proteins are released by alternate, poorly characterized secretory pathways, and would therefore be missed by searching for secretory peptide sequences, as was shown recently for *B. malayi *secreted proteins [23]. In addition, we found more than 700 transcripts with signal peptides from the transcripts that have neither *B. malai *nor *C. elegans *homologs (data not shown).

### Intra-population polymorphism, Synonymous/non-synonymous Single Nucleotide Polymorphism and positive selection

Due to their high evolutionary rates [24,25], nematodes are believed to have a significant number of single nucleotide polymorphisms (SNPs). A large number of sites with SNP (76,568) were detected over the total 23,038,913 assembled bases (total length of the 48,326 transcripts). Since the average coverage of our transcripts is about 10×, we estimated θ (= 4Nμ) for *A. caninum *as 1.2 × 10^-3^. Among the SNP sites only 345 sites have more than two alleles. We obtained confident translations for 6,502 cDNAs containing 20,715 of the 76,568 SNPs. Of these, 10,848 were non-synonymous, and 9,867 were synonymous, with an average dN/dS ratio of 0.3. Among the 518 transcripts possessing more than 9 polymorphic sites, 39 were under positive selection (dN/dS > 1.0), and there were two functional categories identified by the associated GO terms: GO:0004298 (threonine endopeptidase activity and GO:0006511 (ubiquitin-dependent protein catabolic activity). Of these, one parasitism related transcript (contig43771 encoding a protein histidine kinase) exhibited more than 9 polymorphic sites and was under positive selection.

## Discussion

The *A. caninum *transcriptome was sequenced with unprecedented coverage in the present study. While fragmentation is still an issue, the non-biased cloning-free transcript sampling using the Roche/454 technology combined with the conventional Sanger technology in this study enabled an in depth sampling of over 93% of the *A. caninum *transcriptome. Comparing the *A. caninum *transcriptome with the coding sequences of *C. elegans *and *B. malayi *confirmed the high diversity of nematode transcriptomes. Intra-species studies revealed high expression dynamics of the nematode transcriptome, and suggested an impact of the adaptation to parasitism on *A. caninum *genes and gene expression.

Nematodes have higher evolutionary rate than most other eukaryotes [24,25]. Only 20% of our *A. caninum *transcripts shared homology to *C. elegans *genes. Since *A. caninum *and *C. elegans *are from the same phylogenetic clade [12], this lowly shared homology clearly illustrates a high evolutionary rate. The high rate can lead to high polymorphism within species. Our estimation of θ for *A. caninum *is 1.2 × 10^-3^, which is about 2 times higher than that of human coding regions [26]. We may have significantly underestimated θ because each final transcript is probably derived from multiple individuals rather than single worms. This high DNA polymorphism is in agreement with the high evolutionary rate of *A. caninum*. Most SNPs are di-allelic just as detected in human, which suggest a similar mechanism shaping SNPs in both human and nematodes. High mutation rate and diversity are features of the phylum Nematoda. A previous survey of more than 30 nematode species distributed over four nematode clades found that only about 15% of sequenced ESTs could be found in all four nematode clades [27]. The same study suggested that about 30-50% of nematode genes are species specific. Interestingly, our previous studies [16,17], based on limited number of *A. caninum *genes (9,000 and 4,000 genes respectively), found that about 50% of the *A. caninum *genes had homologs in *Caenorhabditis *species. This discrepancy is likely because the conserved genes tend to be expressed at higher levels and therefore are sequenced more deeply. The previous transcriptome studies analyzed transcripts generated using conventional cDNA libraries (cloning based with capillary sequencing), therefore only the most abundant transcripts were represented in those studies. In fact, when only considering the highly expressed transcripts (at least 10 reads sequenced), the homologous rate between *A.caninum *and *C. elegans *is 43%.

The high evolutionary rate and diversity of nematodes may contribute to their ability to adapt to nearly every habitat on earth [28]. In addition, parasitism has evolved independently at least nine times in nematodes [29], and the evolution of parasitism plays a role in shaping the nematode transcriptome. The comparative genomic analysis showed that significantly more *B. malayi *coding genes share homology with *A. caninum *than with *C. elegans*. *B. malayi *is in clade III and both *A. caninum *and *C. elegans *are in clade V, thus it is expected that *B. malayi *share similar homologs with *A. caninum *and *C. elegans *without parasitism adaptation. Parasitic nematodes originated from non-parasites, with subsequent adaptation to the host environment. One major difference between free-living and host environments is the availability and abundance of oxygen. Intestinal parasites like hookworms must adapt to the low oxygen levels in their host by using alternative energy and metabolism pathways. They also need to develop systems to evade the host defense mechanisms. The enriched GO terms of the *A. caninum *transcripts that have homology with *B. malayi *genes but not with *C. elegans *reveal the effect of these adaptations. The most significantly enriched GO term is prolyl oligopeptidase activity. Prolyl oligopeptidase is a family of serine-type endopeptidases [30]. One of its members in the parasitic kinetoplastid, *Trypanosoma cruzi*, is critical for the parasite to invade mammalian host cells [31]. Another highly represented transcript is malic enzyme. Malic enzyme converts malate to pyruvate in the mitochondrion, and is important for adaptation to low oxygen environment in the host [32]. In addition, transcripts encoding nucleic acid binding and DNA binding proteins were also significantly enriched. It is possible that parasitic nematodes interfere with the host's transcription and translation system during invasion, or these transcripts encode endogenous enzymes required for further development and morphological changes that occur in the host. In contrast to the GO term enrichment of these *A. caninum *transcripts, the *B. malayi *genes sharing homology with *C. elegans *only did not exhibit any GO term enrichment.

Despite the strong adaptive capability of nematodes, we failed to find evidence of strong positive selection in *A. caninum*. Only 7.5% of transcripts are under possible positive selection by the dN/dS test. We also failed to detect an extensive positive selection for the parasitism related genes. The lack of extensive positive selection in nematodes could suggest that these organisms have a high mutation rate. We expect our on-going nematode genomic projects to provide additional information about nematode evolution http://www.genome.gov/10002154.

Gene expression in *A. caninum *is highly dynamic, with only half of the genes being constantly expressed over all four stages. This 50% is actually a conservative estimation because we excluded the transcripts that lacked sequencing depth. The most drastic difference is found among expression profiles of larvae and adults, indicating the strong effect of adaptation to the host environment. This is confirmed by the functional GO term enrichments. Genes selectively expressed in parasitic adults (both in F and M) are enriched in functions important for parasitism. In fact, 10 out of the 14 GO terms enriched in parasitic adults are related to parasitism. Cysteine endopeptidase is indispensable for parasites, and its numerous functions enabling parasites to defend against their hosts are well-documented [20,21]. Cysteine proteases are also important for digestion of protein and hemoglobin in the blood meal [33]. Astacin plays a crucial role in *A. caninum *tissue migration [22]. The cuticle is a protective external layer of nematodes that provides the primary defense for parasitic nematodes. Several cuticlar changes associated with parasitism have been documented [34]. The glyceraldehyde-3-phosphate dehydrogenase enzyme is necessary for parasites to utilize host glucose as an energy source. In contrast, genes selectively expressed in larvae tend to be enriched in functions related to basic cellular functions such as oxidoreductase activity, signal transduction, and ribosome structure. The most significant term (based on P-value of GO enrichment test) is rhodopsin-like receptor activity, which are chemosensory receptors in *C. elegans *and may be required for larval nematodes to interact with their free-living environment.

Surprisingly, only a small number of genes were turned on by serum stimulation, which indicate that exposure to host-like conditions in vitro does not trigger immediate gene expression changes. Also, serum stimulation did not turn on expression of an increased number of parasitism related genes in our study. This suggests that hookworms do not drastically change gene expression during the transition to parasitism upon entering their hosts, i.e. many of the molecules that are released during infections are already synthesized in the iL3 and stored for rapid release during infection [35-37]. However, using differential subtractive hybrization, Datu et al [18] found that the genes expressed in serum stimulated *A. caninum *L3 did not overlap with genes associated with developmental changes during recovery of *C. elegans *dauers, and suggested that genes expressed in response to activation with serum were involved in parasitism rather than development [18]. One transcript selectively expressed in ssL3 stage in our study encoded triacylglycerol lipase, which is known to play a role during dauer recovery in *C. elegans *[38]. Invading hookworm L3 have been compared to recovering dauer larvae [39]. Another group of transcripts expressed selectively in ssL3 versus iL3 are the allergen V5/Tpx-1 related proteins or *Ancylostoma *secreted proteins (ASPs), originally isolated from excretory/secretory products of *A. caninum *L3 [35], and subsequently from numerous other nematodes. While its function is unknown, a second, related set of ASPs have been described from the adult stages [40], and were among the most abundant transcripts in adult M and F in this study (Table 3). Overall, expression dynamics correlated with progression through the stages of the life cycle.

Genes with different evolutionary conservation exhibit different expression pattern. Genes conserved among nematodes tend to be expressed consistently in all stages, and *A. caninum *specific genes tend to be expressed more selectively. These expression patterns can be associated with their corresponding functions. Conserved genes are involved in basic cellular activities and thus are required for all stages. Species-specific genes are functionally more closely related to the specific life cycle stage and niche. For example, the iL3 selective transcripts are mainly *A. caninum *specific and are depleted of primary metabolic process (GO:0044238) and macromolecule metabolic process (GO:0043170) (data not shown). In addition, the parasitism related genes are more like to be selectively expressed in adult stages. One would expect this since the adults are parasitic whereas the infective larvae are usually free-living. We found that the male adult differentially expressed transcripts are enriched in serine-type endopeptidase inhibitor activity. The male reproductive tract of mammals is enriched in peptidase inhibitors that function in protection and regulation of fertilization [41], suggesting a similar mechanism may be at work in nematodes.

## Conclusion

In summary, this study allowed generation and cataloging of all genes expressed in four transcriptomes of *A. caninum*. Our analysis segregated those genes in multiple dimensions including functional, developmental and phylogenetic categories. The observations agreed with, but also extended, information on previously described genes, suggesting that the newly discovered genes will provide additional unique value. This information identified sets of stage-specific genes, as well as pre-parasitic and parasitic genes that defined differences in metabolic and cellular processes between stages. Furthermore, parasitic adaptation has been shown to be related to transcriptome diversity and developmental dynamics. This dataset is a resource for more complete microarrays, RT-PCR, RNA interference and proteomics. The latter can identify parasite proteins that occur in specific developmental stages, parasite excretory-secretory products, and the external cuticular surface. In addition, the cDNAs generated will enable better annotation of the upcoming genome sequence http://www.genome.gov/10002154. Such extended genomic studies will aid in the identification of genes involved in host recognition, infection, migration and immune invasion as well as the categorization of targets for vaccine and anthelmintic drugs. Finally, the methodology developed in this study illustrates the effectiveness of deep sequencing as a means for analyzing differential gene expression.

## Methods

### Nematode extraction

The Baltimore strain of *A. caninum *(U.S. National Parasite Collection accession 100655.00) was maintained in beagles as described [42]. Animals were housed and treated in accordance with George Washington University Institutional Care and Use Committee guidelines. Infective L3 (iL3) were recovered from 7-10 day old coprocultures using a modified Baermann technique, washed clean of debris with BU buffer (50 mM Na2PO4/22 mM KH2PO4/70 mM NaCl, pH 6.8; Hawdon et al., 1991), and treated with 1% HCl in BU for 30 min at 22°C. The larvae were washed twice with sterile BU and snap-frozen by immersion in liquid N_2_. Frozen larvae were stored at -80°C until used for library construction. Activated (serum stimulated) larvae were generated as described previously (Brand et al, 2004). Briefly, approximately 5,000 *A. caninum *L_3 _were incubated in a 500 μl volume of RPMI_1640 _medium supplemented with 25 mM HEPES (pH 7.0) and antibiotics (RPMI-complete) containing 10% canine serum filtrate (<10 kDa ultrafiltrate) and 15 mm S-methyl-glutathione (Sigma Chemical). Negative-control (non-activated) L3 were incubated in RPMI-c alone. L3 were incubated in 10% CO_2 _at 37°C for 24 hours. Following incubation, the medium containing the L3 was collected, transferred to microcentrifuge tubes and centrifuged for 5 minutes at 14,000 rpm. The supernatant was removed, and an aliquot of L3 was tested for activation as described previously [43]. L3 pellets showing greater than 85% feeding were pooled and used to isolate nucleic acids. Adult male and female were collected from intestines of infected dogs following euthanasia, and frozen at -80 C until nucleic acids were isolated.

### Preparation of *A. caninum *staged RNA and cDNA libraries

Frozen worm pellets were pulverized using an Alloy Tool Steel Set (Fisher Scientific International). Total RNA from adult and larval parasites was prepared using TRIzol Reagent (GibcoBRL, Life Technologies or Invitrogen, Carlsbad, CA). cDNA libraries from four stages, infective L3 larva (iL3), activated L3 larva (ssL3), adult male (M), and female (F), were generated as previously described [17,44].

### Capillary and high throughput sequencing

The cDNA libraries from the four stages were sequenced using the Roche/454 FLX platform [44] and capillary based Sanger sequencing using ABI 3730 and 3700 platforms [17]. All sequences were deposited to GenBank: accession numbers of the Sanger sequences are BM077300 - BM077991; EW741128 - EW744730; EX534506 - EX567272; EX827505 - EX828593; EY458148 - EY473938; FC539038 - FC555743; the Roche/454 SFF files can be found in SRA, libraries SRX000115-SRX000118. The sequences from different platforms underwent different methods for base calling and detection of high quality regions, trimming of linkers, screening for low complexity regions and contaminants, and returning high-quality sequences. Raw cDNA sequences were processed i.e. quality trimmed and screened for vector sequences using SeqClean http://compbio.dfci.harvard.edu/tgi/. The hybrid assembly was dominated by FLX reads (sff format files), therefore we used the 454 Life Sciences' Newbler assembler v1.1.03.21 followed by addition of the ABI reads. All subsequent analyses were based on these contigs, hereafter referred as transcripts, and their constituent reads.

### Comparative analysis and functional assignments

The core eukaryotic genes (CEGs)[45] were used to estimate the completeness of the *A. caninum *transcriptome. A hidden Markov model profile search of the 48,326 transcripts against the 248 CEG profiles of *C. elegans *genes was carried out using the HMMER [46]. Significant hits were identified according to the suggested cutoffs [45].

*C. elegans *and *B. malayi *coding genes were downloaded from Biomart [47]. The *A. caninum *transcripts were compared against these coding genes using WU-BLAST to identify homologs and matches with a raw BLAST bitscore larger than 50 were considered significant homologs [27]. By this way, *A. caninum *transcripts could be classified in 4 groups: those sharing homology with both *C.elegans *and *B. malayi *genes, those sharing homology with only *C. elegans *genes or only *B. malayi *genes, and those sharing no homology with any other species. We defined the first group as nematode conserved, the last group as *A. caninum *specific, and those sharing homology only with *B. malayi *genes as parasitism related. KEGG orthology (KO)[48] of the transcripts were identified through BLAST searching against the KEGG database. As with the homologs, bitscores larger than 50 were used as a cut-off. The recorded KOs were mapped to the cellular and biochemical pathways using the KEGG reference maps [48]. The number of shared and unique KOs for each pathway was compared and statistically evaluated by Chi-square test with Bonferroni correction.

Gene Ontology (GO) associations of the parasitism related transcripts were performed using Interproscan [49], and significant enrichment of GO terms were computed based on the hypergeometric distribution using FUNC [50]. A probability refinement was done to remove the GO terms identified as significant due to their children terms. GO term enrichment analysis is vulnerable to false discovery. We used the false discovery rate (FDR) computed by FUNC to reduce false discovery. Therefore, unless specified otherwise, the GO term enrichment was selected based on both p-value < 0.05 (after refinement) and FDR <0.1.

### Expression pattern examination

Expression patterns were defined by stage specificity and stage bias of the transcript's constituent reads. After assembly, transcripts with length greater than 90 base pairs (bp) were subjected to expression pattern examination as follows: the numbers of reads originating from the different cDNA libraries for each transcript was recorded, and transcripts with reads originating from only one stage (or a set of stages) were designated to be specific to that stage (or that set of stages). To increase the confidence with which specificity was assigned, we required the transcripts to be sequenced deeply enough to ensure that an observed stage absence (i.e. 0 reads from a specific stage) have a confidence interval above 95%. It turned out that this required the transcript be sequenced at least 10 times (i.e. transcripts have at least 10 constituent reads). Specifically, for each transcript, its observed absence from a stage (if any) was compared to its expected number of stage-specific reads (calculated by multiplying the marginal read frequency of the four stages by the total number of reads in that transcript). If the probability of the observed absence (to a Poisson distribution with the mean as the expected number of reads) was higher than 0.05, the transcript was considered to lack depth of coverage and was excluded from the analysis (including the function-related analyses). This requirement excluded 32,099 transcripts, leaving 16,359 transcripts with defined stage-specificity (with high confidence).

Stage-specific expression implies that a specific gene is expressed during one developmental stage (or one set of development stages) but not in the other. However, gene expression does not always follow the on/off model. Its expression dynamics also includes the expression fluctuation in different stages. In some stages one gene may have enhanced expression while it may have depleted expression in other stages. Such transcripts were designated as stage-biased in our analysis. These transcripts that were biased in expression towards a certain stage were selected by comparing the numbers of reads in different stages (iL3, ssL3, and Adult) using a statistical approach defined by Audic et al. [51] with a significance of P < 1e-05. We focused on the comparisons of iL3 vs ssL3 and ssL3 vs Adult (male and females were treated together as adult stage).

### Functional examination of transcripts with different expression pattern

The predicted functions of the transcripts with different expression patterns were examined with GO association as described previously. In consideration of the amount of data and parasitism, we focused on 5 groups of transcripts: those constantly expressed in all four stages; those selectively expressed in both iL3 and ssL3; those exclusively expressed in F, those exclusively expressed in M; and those selectively expressed in both F and M. To visualize the functional profile of these groups, we used heatmap to illustrate the enrichment and depletion of GO terms related to molecular function. To increase the visibility, we included only the GO terms that are enriched or depleted in at least one of the 5 groups. Meanwhile, significantly enriched GO terms in molecular function of these groups were recorded and manually examined to explore the functional association of selective expression.

### Identification of secretory parasitism related genes

Upon the identification of parasitism related genes, several criteria were required to further identify parasitism genes encoding secretory proteins [52] that the parasite might use to interfere with the host cellular functions and enable successful parasitism. These criteria require the genes i) to share homology to *B. malayi *genes but not *C. elegans *(are in the subset of parasitism related genes identified above), ii) are expressed in the parasitic stages (ssL3, M, F) but should not be expressed in the preparasitic iL3 stage; iii) to have signal peptide for secretion (signal peptides for secretions and trans-membrane domains were identified using PHOBIUS [53], with an additional requirement that the SP is within the first 70 amino acids). Hence, the transcripts expressed in parasitic stages, without homologs in *C. elegans *but with a significant homology to *B. malayi *genes and with a signal peptide for secretion but without membrane containing domains were classified as putative secretory parasitism genes. The functions of these transcripts were assigned based on homology found by InterProScan [49].

### Intra-population polymorphism and synonymous and non-synonymous Single Nucleotide Polymorphisms (SNPs)

The cDNAs generated in this study originate from RNA isolated from a population of individuals, enabling us to estimate the rate of population polymorphism of the transcriptomic reads. We used Polybayes [54] to detect the SNPs by using the transcript (i.e. contigs) as reference. Based on the detected SNPs, we estimated the θ, a representation of DNA sequence polymorphism which is related to the mutation rate and effective population size, by  following Watterson [55], where n = 10 (the estimated coverage depth) and S is the fraction of sites with polymorphism. By treating the estimated coverage depth as n, we have likely underestimated θ. To identify if the SNPs contribute to synonymous or non-synonymous changes the transcripts were translated using Prot4Est [56]. The dN and dS were calculated using the method of Nei and Gojobori [57]. To improve reliability of the estimation, only transcripts with more than 9 polymorphic sites were analyzed. Functions of transcripts under positive selection (dN/dS > 1.0) were further investigated for GO term enrichment (as stated above).

## Abbreviations

L3: third stage larvae; iL3: infective L3; ssL3: serum stimulated L3; F: female; M: male; Ad: adult; cDNA: complementary DNA; GO: Gene Ontology; KEGG: Kyoto Encyclopedia of Genes and Genomes; KO: KEGG Ontology; SNP: Single Nucleotide Polymorphism; dN: non-synonymous polymorphism; dS: synonymous polymorphism.

## Authors' contributions

ZW and MM conceived and designed the experiments. JH provided the worms/RNA. JM, SA and ZW carried out experiments and analyses. ZW, RKW, JH and MM interpreted results and prepared the manuscript. All authors have read and approved the final manuscript.

## Supplementary Material

Additional file 1 Coverage of the *A. caninum *transcriptome based on low copy number conserved eukaryotic genes. Click here for file

Additional file 2 The top 20 most abundant stage-specific and constantly expressed *A. caninum *genes. Click here for file

Additional File 3 **Stage-biased differential expression of *A. caninum *genes.** A). Comparison among preparasitic and parasitic larval stages, and parasitic larval and adult stage. B) Comparison of expression profiles generated by suppression subtractive hybridization and cDNA sequencing of stage-specific libraries. Click here for file

Additional file 4 Functional enrichment and depletion score derived from the enrichment and depletion probabilities calculated by hypogeometrics test of molecular functional GO terms. Click here for file

Additional file 5 The top 10 most abundant stage-specific and constantly expressed *A. caninum *genes having homology to *B. malayi *but not *C. elegans*. Click here for file

Additional file 6 Putative *A.caninum *parasitic genes and their functions. Click here for file
